# Re-emergence of invasive pneumococcal disease (IPD) and increase of serotype 23B after easing of COVID-19 measures, Switzerland, 2021

**DOI:** 10.1080/22221751.2021.2000892

**Published:** 2021-12-04

**Authors:** Carlo Casanova, Marianne Küffer, Stephen L. Leib, Markus Hilty

**Affiliations:** a Swiss National Reference Center for Invasive Pneumococci (NZPn), Institute for Infectious Diseases, University of Bern, Bern, Switzerland; b Institute for Infectious Diseases, University of Bern, Bern, Switzerland

**Keywords:** *Streptococcus pneumoniae*, surveillance, COVID-19 restrictions, non-susceptibility to penicillin, pneumococcal conjugate vaccine (PCV)

## Abstract

Incidence of invasive pneumococcal disease (IPD) has been low during the peak of the COVID-19 pandemic. In this study, we found that the IPD numbers again increased in Switzerland during the first six months of 2021 and that this coincides with the loosening of COVID-19 measures. Vaccine pneumococcal serotypes have continued to decrease and non-vaccine type serotype 23B has emerged (8% of the isolates in 2021). Worryingly, serotype 23B is associated with reduced susceptibility to penicillin.

## Introduction

Before the onset of the COVID-19 pandemic, the Swiss National Center for Invasive Pneumococci (NZPn) noted a record number of IPD isolates in Switzerland in 2008 (*n* = 1129) which then steadily decreased to 877 isolates in 2016 [1]. Thereafter, numbers remained constant (*n* = 915 in 2019), which roughly translates to 10 cases per 100,000 inhabitants. During the first months of the COVID-19 pandemic, we and others discovered a drop in the incidence of IPD within the Invasive Respiratory Infection Surveillance Initiative which performed a prospective analysis of surveillance data from 1 January 2018, to 31 May 2020 [2]. More information is now eagerly awaited as to how the IPD cases will evolve once the different countries start to overcome the pandemic and more and more reduce lockdown restrictions.

In this study, we, therefore, aimed at analysing the serotype epidemiology and antibiotic resistance profiles of IPD isolates from Switzerland up to the end of June 2021.

## Method

In Switzerland, it is mandatory for all clinical microbiology laboratories to send IPD isolates from sterile body sites to the NZPn. We estimate that roughly 90% of isolates can be linked to physician-reported IPD cases [3]. For this study, all the IPD isolates between January 2017 and June 2021 have been serotyped by Quellung reaction. Susceptibility to erythromycin, trimethoprim-sulfamethoxazole and levofloxacin was determined by disc diffusion according to the European Committee on Antimicrobial Susceptibility Testing (EUCAST) and, prior to 2018, according to The Clinical & Laboratory Standards Institute (CLSI) [1,4]. Reduced penicillin susceptibility detected by oxacillin disc screen was confirmed by Etest (bioMérieux, Marcy-l’Étoile, France) applying meningitis interpretation criteria.

## Results

**Figure 1.**
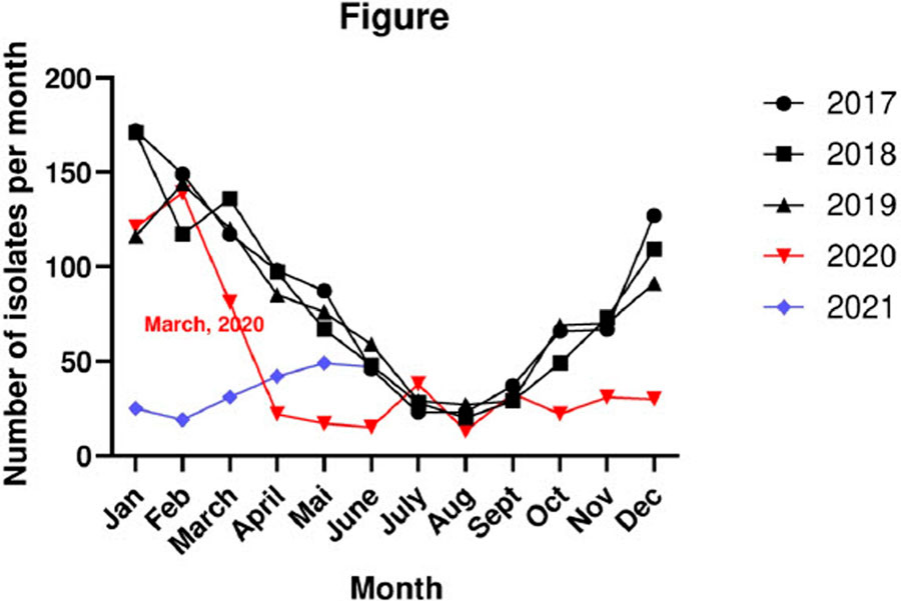
Number of invasive pneumococcal disease (IPD) isolates in Switzerland, January 2017–June 2021. Shown are the total numbers per month. The data for 2020 and 2021 are indicated in red and blue, respectively. The first month with COVID-19 measures is labeled (March 2020).

We received 1012, 944, 915, 562 and 213 IPD isolates in 2017, 2018, 2019, 2020 and the first half year of 2021, respectively (Figure 1). From February 2020 (*n* = 139) to April 2020 (*n* = 22), we observed a drastic decline of IPD isolates, probably due to the COVID-19 measures. Numbers then remained low from April 2020-February 2021 (*n* = 19). Strikingly, numbers started to increase from March 2021 (*n* = 31) – Mai 2021 (*n* = 49). By June 2021, we again registered approximately the same number of IPD isolates (*n* = 47) as for June 2019, June 2018 and June 2017 (i.e. before the COVID-19 measures).

The observed rebound in IPD isolates coincided with the fact, that the COVID-19 measures have been loosened by the Swiss Government on 1 March 2021. Shops, museums and libraries were allowed to reopen and outdoor sports in small groups up to 15 people took again place. In addition, young people (born in 2001 or after) were again allowed leisure activities including (indoor) sports, culture and singing (including choir) (https://www.bag.admin.ch/dam/bag/en/dokumente/mt/k-und-i/aktuelle-ausbrueche-pandemien/2019-nCoV/tabelle-aenderungen-massnahmen.pdf.download.pdf/Changes_measures.pdf).

For the first half year of 2021, 5.6% and 19.2% were of pneumococcal conjugate vaccine PCV7 and PCV13 non-PCV7 serotypes, respectively (Supplementary Table 1). The proportion of PCV13 serotypes has thus further decreased as compared to the years before the COVID-19 pandemic (2017–2019). Most apparent, only three isolates (1.4%) with PCV7 serotypes were detected for the ≥65-year-old patients in the first six months of 2021 as compared to 26 in 2020 (4.6%; *P* = .04 Chi-square test) (Supplementary Table 1).

As for individual PCV13 serotypes, serotypes 19A, 19F and 3 were found in >3% of all the isolates for the first half year of 2021 (Supplementary Table 2). The proportion of serotype 3 has been high in the years before COVID-19 measures and has not yet declined in 2021 (15.5% of the isolates). However, most strikingly, the proportion of non-vaccine serotype 23B increased in 2021 (8.0%) as compared to 2020 (3.2%). This increase was found to be significant (*P* = .004; Chi-Square test), and serotype 23B has now been identified as the second most frequent non-vaccine serotype in our study. The proportions of the other non-vaccine serotypes remained roughly unchanged in 2021 as compared to 2020.

Eighteen of the 213 IPD isolates in the first half of 2021 were non-susceptible to penicillin (8.5%). Compared to previous years, the resistance rate (meningitis criteria) thus slightly increased (7.4% in 2019, 7.8% in 2020). Importantly, more than half of the penicillin-resistant isolates in 2021 were of serotype 23B (55.6%) (Supplementary Figure 1). The penicillin minimal inhibitory concentrations (MICs) of these isolates were only moderately elevated (range 0.064–0.19 mg/L) and were thus still in the intermediate range (“susceptible, increased exposure”) for indications other than meningitis. All of the 23B serotype isolates analyzed in 2021 so far were susceptible to ceftriaxone, erythromycin and levofloxacin (the latter “susceptible, increased exposure”). Six of the 10 penicillin-resistant isolates were, however, also non-susceptible to trimethoprim-sulfamethoxazole.

## Discussion

The 13-valent pneumococcal conjugate vaccine (PCV-13) became the recommended pneumococcal vaccine in Switzerland in 2014 and is now widely used (vaccine coverage rates are >90% for children below two years). Therefore, and as noted in other countries, a decrease in PCV13 serotypes is not surprising [1,5]. In 2019, we observed that only 29.5% of the isolates still were PCV13 serotypes of which serotype 3 was the most frequent (16.3%). During the COVID-19 pandemic, a drop in the incidence of IPD has been observed in Switzerland and all over Europe [2]. It is now of great interest to explore how COVID-19 measures and the loosening thereof affect the epidemiology of IPD.

In this study, we analysed the total number of IPD isolates and the proportion of PCV13 serotypes among them. As a limitation, we do not report denominator data as these are not yet available for 2020–2021 in Switzerland. We found that PCV13 serotypes have further decreased but that there is a high proportion of penicillin non-susceptible *S. pneumoniae* of serotype 23B.

A very recent study also determined the serotype distribution and antimicrobial resistance of *Streptococcus pneumoniae* associated with mucosal infections in young children [6]. Very similar to our study, they found that isolates exhibiting serotype 23B were often penicillin non-susceptible (38%) [6]. In Portugal, it has been shown that a high proportion of penicillin non-susceptible isolates was of serotype 23B in IPD in adults and that 23B was associated with isolation from cerebrospinal fluid samples [7].

In this respect, it is perhaps also of relevance that serogroup 23 (excluding 23F) has been identified as one of three emerging, non-PCV13 serotype in a Swiss study analysing pneumococcal carriage in the nasopharynx of children with acute otitis media [8]. Similarly, 23B was also among the most prevalent non-PCV13 serotypes in more recent years in patients with otitis media in Germany [9]. Interestingly, penicillin-resistant *S. pneumoniae* of serotype 23B has also found to be the most frequent non-vaccine serotype in a carriage study in Ghana [10]. Taken together, this illustrates that carriage data is also urgently needed to better understand the overall epidemiology of *S. pneumoniae*.

In conclusion, we found that the number of IPD isolates has again increased to a similar level as before the implementation of COVID-19 measures in Switzerland. Also, penicillin MICs of isolates of serotype 23B are elevated, which is worrying as this is also the serotype that we found has significantly increased in 2021. For the future direction, a careful, continuous monitoring of the circulating serotypes is of uttermost importance, as more and more countries are easing the COVID-19 measures.

## Supplementary Material

Supplementary_tablefigures_R1_vf.docx

## References

[1] Oyewole OR, Lang P, Albrich WC, et al. The impact of pneumococcal conjugate vaccine (PCV) coverage heterogeneities on the changing epidemiology of invasive pneumococcal disease In Switzerland, 2005-2019. Microorganisms. 2021;9(5), 1078. 34069761 10.3390/microorganisms9051078 PMC8157260

[2] Brueggemann AB, van Rensburg MJ J, Shaw D, et al. Changes In the incidence of invasive disease due to Streptococcus pneumoniae, haemophilus influenzae, and neisseria meningitidis during the COVID-19 pandemic In 26 countries and territories In the invasive respiratory infection surveillance initiative: a prospective analysis of surveillance data. The Lancet Digital Health. 2021;3(6):e360–e70. 34045002 10.1016/S2589-7500(21)00077-7 PMC8166576

[3] Meichtry J, Born R, Kuffer M, et al. Serotype epidemiology of invasive pneumococcal disease In Swiss adults: a nationwide population-based study. Vaccine. 2014;32(40):5185–5191. 25077419 10.1016/j.vaccine.2014.07.060

[4] Hauser C, Kronenberg A, Allemann A, et al. Serotype/serogroup-specific antibiotic non-susceptibility of invasive and non-invasive *Streptococcus pneumoniae*, Switzerland, 2004 to 2014. Eurosurveillance. 2016;21(21):12–21. 27254535 10.2807/1560-7917.ES.2016.21.21.30239

[5] Knoll M D, Bennett JC, Garcia Quesada M, et al. Global landscape review of serotype-specific invasive pneumococcal disease surveillance among countries using PCV10/13: the pneumococcal serotype replacement and Distribution estimation (PSERENADE) project. Microorganisms. 2021 Apr 2;9(4):742. 33918127 10.3390/microorganisms9040742 PMC8066045

[6] Udden F, Runow E, Slotved HC, et al. Characterization of *Streptococcus pneumoniae* detected In clinical respiratory tract samples In southern Sweden 2 to 4 years after introduction of PCV13. J Infect. 2021 Aug;83(2):190–196. 34062179 10.1016/j.jinf.2021.05.031

[7] Silva-Costa C, Gomes-Silva J, Teodoro I, et al. Invasive pneumococcal disease in adults in Portugal: the importance of serotypes 8 and 3 (2015–2018). Microorganisms. 2021;9(5), 1016. 34066862 10.3390/microorganisms9051016 PMC8150758

[8] Allemann A, Frey PM, Brugger SD, et al. Pneumococcal carriage and serotype variation before and after introduction of pneumococcal conjugate vaccines in patients with acute otitis media in Switzerland. Vaccine. 2017;35(15):1946–1953. 28279564 10.1016/j.vaccine.2017.02.010

[9] Imohl M, Perniciaro S, Busse A, et al. Bacterial spectrum of spontaneously ruptured otitis media In a 7-year, longitudinal, multicenter, epidemiological cross-sectional study In Germany. Front Med (Lausanne). 2021;8:675225. 34095179 10.3389/fmed.2021.675225 PMC8172772

[10] Mills RO, Abdullah MR, Akwetey SA, et al. Post-Vaccination *Streptococcus pneumoniae* carriage and virulence gene distribution among children less than five years of age, cape coast, Ghana. Microorganisms. 2020;8(12), 1987. 10.3390/microorganisms8121987 PMC7764876 33322236

