# Supplementary material for: Re-emergence of invasive pneumococcal disease (IPD) and increase of serotype 23B after easing of COVID-19 measures, Switzerland, 2021
Source: Emerg Microbes Infect. 2021 Dec 4;10(1):2202–4. doi: 10.1080/22221751.2021.2000892 (PMC8648035; doi:10.1080/22221751.2021.2000892)
Supplement: Supplementary_tablefigures_R1_vf.docx [file TEMI_A_2000892_SM3889.docx]

**Supplementary table 1**. Proportions of PCV serotypes among invasive pneumococcal disease cases in Switzerland, January 2017- June 2021

| **Age category** | **Serotype proportions of IPD cases in age groups (%)** | | | | |
| --- | --- | --- | --- | --- | --- |
|  | **Year** |  |  |  |  |
|  | **2017** | **2018** | **2019** | **2020** | **2021** |
| < 5 years | 3 (0.3) | 3 (0.3) | 1 (0.1) | 1 (0.2) | 0 (0) |
| 5-64 years | 36 (3.6) | 31 (3.3) | 33 (3.6) | 18 (3.2) | 7 (3.3) |
| ≥65 years | 41 (4.1) | 38 (4) | 44 (4.8) | 26 (4.6) | 3 (1.4) |
| Unknown | 5 (0.5) | 1 (0.1) | 1 (0.1) | 2 (0.4) | 2 (1.0) |
| **Total *PCV7^a^*** | 85 (8.4) | 73 (7.7) | 79 (8.6) | 47 (8.4) | 12 (5.6) |
|  |  |  |  |  |  |
| < 5 years | 7 (0.7) | 18 (1.9) | 7 (0.8) | 2 (0.4) | 3 (1.4) |
| 5-64 years | 74 (7.3) | 79 (8.4) | 58 (6.3) | 40 (7.1) | 16 (7.5) |
| ≥65 years | 146 (14.4) | 130 (13.8) | 110 (12) | 78 (13.9) | 19 (8.9) |
| Unknown | 24 (2.4) | 15 (1.6) | 16 (1.7) | 8 (1.4) | 3 (1.4) |
| ***Total PCV13nonPCV7^b^*** | 251 (24.8) | 242 (25.6) | 191 (20.9) | 128 (22.8) | 41 (19.2) |
|  |  |  |  |  |  |
| < 5 years | 17 (1.7) | 17 (1.8) | 16 (1.7) | 15 (2.7) | 6 (2.8) |
| 5-64 years | 215 (21.2) | 203 (21.5) | 207 (22.6) | 143 (25.4) | 57 (26.8) |
| ≥65 years | 391 (38.6) | 359 (38) | 375 (41) | 187 (33.3) | 85 (40.0) |
| Unknown | 53 (5.2) | 50 (5.3) | 47 (5.1) | 42 (7.4) | 12 (5.6) |
| ***Total Non-PCV^c^*** | 676 (66.8) | 629 (66.6) | 645 (70.5) | 387 (68.9) | 160 (75.1) |
|  |  |  |  |  |  |
| < 5 years | 27 (2.7) | 38 (4.0) | 24 (2.6) | 18 (3.2) | 9 (4.2) |
| 5-64 years | 325 (32.1) | 313 (33.2) | 298 (32.6) | 201 (35.8) | 80 (37.6) |
| ≥65 years | 578 (57.1) | 527 (55.8) | 529 (57.8) | 291 (51.8) | 107 (50.2) |
| Unknown | 82 (8.1) | 66 (7.0) | 64 (7.0) | 52 (9.2) | 17 (8.0) |
| **All cases** | **1012 (100)** | **944 (100)** | **915 (100)** | **562 (100)** | **213 (100)** |
| a Serotypes included in PCV7: 4, 6B, 9V, 14, 18C, 19F & 23F  b Additional serotypes included in PCV13, but not PCV7: 1, 3, 5, 6A, 7F & 19A  c Serotypes not included in either PCV7 or PCV13 | | | | | |

**Supplementary table 2**: Serotype distribution of referred IPD isolates 2017-2021 (2020-2021 data are shown for the first and second 6 months of the year)

|  | **2017** | | **2018** | | **2019** | | **1^st^ 2020** | | **2^nd^ 2020** | | **1^st^ 2021** | |
| --- | --- | --- | --- | --- | --- | --- | --- | --- | --- | --- | --- | --- |
| **Serotype** | n | % | n | % | n | % | n | % | n | % | n | % |
| **PCV7** |  |  |  |  |  |  |  |  |  |  |  |  |
| **19F** | 27 | 2.7 | 18 | 1.9 | 23 | 2.5 | 9 | 2.3 | 5 | 3.0 | 8 | 3.8 |
| **4** | 21 | 2.1 | 13 | 1.4 | 8 | 0.9 | 3 | 0.8 | 3 | 1.8 | 2 | 1 |
| **14** | 17 | 1.7 | 19 | 2 | 22 | 2.4 | 8 | 2.0 | 5 | 3.0 | 0 | 0 |
| **6B** | 9 | 0.9 | 4 | 0.4 | 4 | 0.4 | 4 | 1.0 | 1 | 0.6 | 0 | 0 |
| **18C** | 1 | 0.1 | 6 | 0.6 | 8 | 0.9 | 4 | 1.0 | 0 | 0.0 | 1 | 0.5 |
| **23F** | 4 | 0.4 | 5 | 0.5 | 2 | 0.2 | 4 | 1.0 | 1 | 0.6 | 1 | 0.5 |
| **9V** | 6 | 0.6 | 8 | 0.8 | 12 | 1.3 | 0 | 0.0 | 0 | 0.0 | 0 | 0 |
| **PCV13 non-PCV7** |  |  |  |  |  |  |  |  |  |  |  |  |
| **3** | 158 | 15.6 | 187 | 19.8 | 149 | 16.3 | 71 | 16.9 | 24 | 14.4 | 33 | 15.5 |
| **19A** | 63 | 6.2 | 42 | 4.4 | 31 | 3.4 | 20 | 4.4 | 5 | 3.0 | 7 | 3.3 |
| **7F** | 22 | 2.2 | 9 | 1 | 6 | 0.7 | 3 | 0.7 | 1 | 0.6 | 1 | 0.5 |
| **6A** | 5 | 0.5 | 4 | 0.4 | 4 | 0.4 | 2 | 0.7 | 2 | 1.2 | 0 | 0 |
| **1** | 3 | 0.3 | 0 | 0 | 1 | 0.1 | 0 | 0.0 | 0 | 0.0 | 0 | 0 |
| **5** | 0 | 0 | 0 | 0 | 0 | 0 | 0 | 0.0 | 0 | 0.0 | 0 | 0 |
| **Total PCV13** | 336 | 33.2 | 315 | 33.4 | 270 | 29.5 | 128 | 32.4 | 47 | 28.1 | 53 | 24.9 |
| **8** | 150 | 14.8 | 160 | 16.9 | 142 | 15.5 | 68 | 17.2 | 28 | 16.8 | 44 | 20.7 |
| **23B** | 39 | 3.9 | 33 | 3.5 | 23 | 2.5 | 11 | 2.8 | 7 | 4.2 | 17 | 8 |
| **22F** | 91 | 9 | 86 | 9.1 | 103 | 11.3 | 31 | 7.8 | 12 | 7.2 | 15 | 7 |
| **9N** | 64 | 6.3 | 50 | 5.3 | 63 | 6.9 | 23 | 5.8 | 10 | 6.0 | 14 | 6.6 |
| **24** | 15 | 1.5 | 13 | 1.4 | 13 | 1.4 | 8 | 2.0* | 4 | 2.4* | 3 | 1.4* |
| **15A** | 31 | 3.1 | 27 | 2.9 | 29 | 3.2 | 9 | 2.3 | 2 | 1.2 | 5 | 2.3 |
| **12F** | 29 | 2.9 | 37 | 3.9 | 48 | 5.2 | 11 | 2.8 | 2 | 1.2 | 4 | 1.9 |
| **10A** | 24 | 2.4 | 25 | 2.6 | 30 | 3.3 | 14 | 3.5 | 5 | 3.0 | 6 | 2.8 |
| **15B/C** | 24 | 2.4 | 25 | 2.6 | 19 | 2.1 | 13 | 3.3* | 8 | 4.8* | 6 | 2.8* |
| **6C** | 22 | 2.2 | 15 | 1.6 | 13 | 1.4 | 8 | 2.0 | 2 | 1.2 | 4 | 1.9 |
| **11A** | 27 | 2.7 | 24 | 2.5 | 19 | 2.1 | 6 | 1.5 | 6 | 3.6 | 8 | 3.8 |
| **23A** | 27 | 2.7 | 20 | 2.1 | 29 | 3.2 | 10 | 2.5 | 4 | 2.4 | 6 | 2.8 |
| **35F** | 15 | 1.5 | 16 | 1.7 | 20 | 2.2 | 6 | 1.5 | 6 | 3.6 | 3 | 1.4 |
| **31** | 18 | 1.8 | 6 | 0.6 | 7 | 0.8 | 5 | 1.3 | 1 | 0.6 | 1 | 0.5 |
| **38** | 5 | 0.5 | 6 | 0.6 | 11 | 1.2 | 5 | 1.3 | 0 | 0.0 | 1 | 0.5 |
| **16** | 15 | 1.5 | 9 | 1 | 11 | 1.2 | 7 | 1.8* | 4 | 2.4* | 2 | 1.0* |
| **20** | 17 | 1.7 | 13 | 1.4 | 12 | 1.3 | 2 | 0.5 | 1 | 0.6 | 3 | 1.4 |
| **33F** | 16 | 1.6 | 16 | 1.7 | 13 | 1.4 | 8 | 2.0 | 3 | 1.8 | 6 | 2.8 |
| **17F** | 8 | 0.8 | 8 | 0.8 | 11 | 1.2 | 1 | 0.3 | 1 | 0.6 | 3 | 1.4 |
| **10B** | 4 | 0.4 | 4 | 0.4 | 3 | 0.3 | 3 | 0.8 | 1 | 0.6 | 1 | 0.5 |
| **35B** | 14 | 1.4 | 11 | 1.2 | 5 | 0.5 | 5 | 1.3 | 5 | 3.0 | 2 | 1 |
| **Other** | 21 | 2.1 | 25 | 2.6 | 21 | 2.3 | 13 | 3.3 | 8 | 4.8 | 6 | 2.8 |
| **Total non-PCV13** | 676 | 66.8 | 629 | 66.6 | 645 | 70.5 | 267 | 67.6 | 120 | 71.9 | 160 | 75.1 |
| **Total** | 1012 | 100 | 944 | 100 | 915 | 100 | 395 | 100 | 167 | 100 | 213 | 100 |

*Serogroup 16 and 24 exclusively consisted of 16F and 24F isolates, 15B/C consisted of 15B (n=12) and 15C (n=9) isolates.

Supplementary Figure: Percentage of pneumococcal serotypes of penicillin non-susceptible IPD isolates in Switzerland
